# Kinematic Analysis of 2-Point and 3-Point Jump Shot of Elite Young Male and Female Basketball Players

**DOI:** 10.3390/ijerph18030934

**Published:** 2021-01-22

**Authors:** Tomas Vencúrik, Damir Knjaz, Tomislav Rupčić, Goran Sporiš, Feng Li

**Affiliations:** 1 Department of Sports, Faculty of Sports Studies, Masaryk University, 62500 Brno, Czech Republic; vencurik@fsps.muni.cz; 2 Laboratory for Sports Games, Faculty of Kinesiology, University of Zagreb, 10000 Zagreb, Croatia; damir.knjaz@kif.unizg.hr (D.K.); tomislav.rupcic@kif.unizg.hr (T.R.); li.feng@kif.unizg.hr (F.L.)

**Keywords:** catch-and-shoot, curl cut, center of mass, shoulder angle, entry angle

## Abstract

Basketball shooting is one of the most important offensive skills in basketball. Winning or losing a game mostly depends on the shooting effectiveness. The study aims to compare the selected kinematic variables of 2-point (2-pt) and 3-point (3-pt) jump shots (after making a cut and receiving the ball) and ascertain the differences between elite male under 16 and 18 (U16M, U18M) and female under 16 and 18 (U16F, U18F) basketball players. Overall, forty-eight young male and female basketball players participated in the study. 3D motion analysis using an inertial suit with the addition of utilizing a smart ball was performed for assessing the 2-pt and 3-pt shooting techniques. Players in male categories shot for 2-pt with a higher center of mass difference in the vertical direction (U16M 5.7 cm, U18M 3.9 cm vs. U16F 1.4 cm, U18F 0.6 cm), with higher release shoulder angle (U16M 110.9, U18M 113.8 vs. U16F 103, U18F 105), and with a higher entry angle of the ball (U16M 34, U18M 32 vs. U16F 30, U18F 30) when compared to female categories (*p* < 0.001). In the 3-pt shooting, there were differences between male and female categories in the shoulder angle when releasing the ball (*p* < 0.001). In the players shooting speed, there were differences between U16M vs. U18F (0.95 ± 0.1 vs. 0.88 ± 0.1; *p* = 0.03) and U16F vs. U18F (0.96 ± 0.06 vs. 0.88 ± 0.1; *p* = 0.02) players. Male categories shot 3-pt shots with a smaller center of mass difference in the horizontal direction when compared to 2-pt shots (*p* < 0.001). The entry angle was higher in successful shooting attempts compared to unsuccessful shooting attempts when shooting for 3-pt (*p* = 0.02). Player shooting speed was higher in all categories (except U18F) when shooting for 3-pt (*p* < 0.001). It appears that performers show difference in kinematic variables based on distance from the basket. Basketball coaches and players should work to minimize the kinematic differences between 2-pt and 3-pt shooting and to optimize the shooting technique.

## 1. Introduction

Basketball shooting is an essential offensive skill because it directly influences the outcome of the game. Other basketball offensive skills such as dribbling and passing are used by players to create the best position for shooting. Several studies confirm the importance of basketball shooting. The investigation into game-related statistics revealed that especially effective field goals (along with defensive rebounds, free throw percentages, and assists) correlated with win/loss in elite basketball competitions [1,2,3]. Field goals include more types of shooting, i.e., lay-ups, jump shots, dunks, hook shots, and tip-ins. Except for jump shots, the other types of shooting are used primarily close to the basket. More than 60% of all field goal attempts in the Women’s National Basketball Association (WNBA) in the 2010 season were jump shots, which supports the importance of this type of shooting [4]. National Basketball Association (NBA) shows similar statistics in shooting. According to NBA.com/Stats [5], 58.8% of all jump shots taken during the regular season 2016–2017, by all NBA teams. Furthermore, 29.5% of all shots were taken after receiving a pass (catch and shoot situation). The most common situation is when the offensive player with the ball drives to the basket and the weak side defender has to help with the drive. Then the player passes the ball to an undefended teammate who can shoot. Therefore, these statistics confirm the importance of shooting after receiving the pass. In this context, Ibáñez et al. [6] based on game-related statistics, detects the dependency between assisted shots and the winning of the game. The team, which made more assists in the game, had a higher chance to win the game. 

In the past, various aspects related to jump shots were investigated. Miller and Bartlett [7] and Okazaki and Rodacki [8] assessed the impact of increasing horizontal distance on the kinematic parameters of a jump shot (segmental joint angle, center of mass displacement, release angle, release speed, etc.). When the distance increases, the player reduces the ball release angle, and the ball follows a flatter flight path. Erčulj and Supej [9] observed the impact of fatigue on joint angles and the height of the jump. The measured elbow and upper arm angles decrease with the growing fatigue. Their study demonstrates significant changes in shooting technique as a consequence of moderate and high fatigue. Another study, Rojas et al. [10] explores whether a defender’s presence has any impact on selected kinematic parameters. When a player is confronted by an opponent, the ball is released faster and from a greater height. Furthermore, some studies investigate the visual control of a jump shot effectivity because this determinant of basketball shooting is considered important [11,12]. Training the visual control forces players to learn to use the visual information about the rim in a shorter time (up to 400 ms). It means that players have more time to perceive other factors that relate to the game.

Scientific literature mentioned above assessing the kinematic and physical parameters of a jump shot presents only shots taken without any action before shooting (dribbling or cutting—no pull-up jump shots or catch-and-shoot jump shots). Measuring kinematic and physical parameters of a jump shot that are more similar to real game conditions is absent (e.g., catch-and-shoot situation after a cut). Moreover, comparing these parameters between gender is even less studied having in mind the differences between male and female in physical performance. The novelty and uniqueness of the current study is in the documented selected kinematic variables of the jump shot after making a cut and receiving a ball. Therefore, this study aims to compare the selected kinematic variables of a basketball jump shot (separately for 2-point and 3-point shots) after receiving the ball (catch-and-shoot situation after a cut) and ascertain the differences between the elite U16 and U18 male and female basketball players, and between successful and unsuccessful shots. It was hypothesized that differences in selected kinematic variables will be observed between 2-point and 3-point shots regarding different categories and success of the shots. 

## 2. Materials and Methods 

### 2.1. Participants

Fifty-eight basketball players participated in this study. Due to some technical issues with equipment and incorrect data, ten subjects were removed from the final data analysis. After removing ten players, forty-eight Croatian elite young basketball players were included in the research. All selected players were chosen for a wider national selection for the 2017 European Championship in U16 and U18 male and female categories. Sixteen male basketball players were members of the U16 team (U16M) with an average age 15.4 ± 0.6, body height 192.6 ± 6.3 cm, and body mass 80.5 ± 9.8 kg. Fourteen male basketball players were members of the U18 team (U18M) with average age 17 ± 0.7, body height 197.9 ± 7.9 cm, and body mass 87.5 ± 9.3 kg. The U16 female team (U16F) included eleven basketball players with average age 15.5 ± 0.9, body height 178.3 ± 5.7 cm, and body mass 69 ± 9.1 kg. The U18 female team (U18F) included seven basketball players with average age 17 ± 0.8, body height 176.5 ± 7.4 cm, and body mass 74.2 ± 8.8 kg. Participants had no injuries that could have affected their shooting performance. Players were informed about this study’s goals, participated voluntarily, and signed the informed consent (in the case of a minor, a legal representative). The study was conducted in accordance with the Declaration of Helsinki and following the ethical standards of the University of Zagreb (ethical approval number: 113/2016). 

### 2.2. Procedure

**Figure 1:**
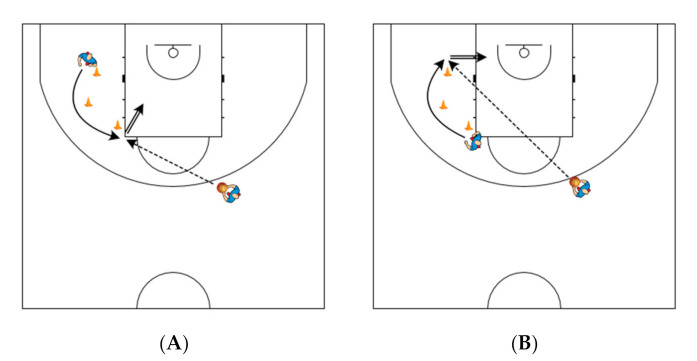
(**A**): 2-pt shooting after curl-cut with the angle of 60° from the backboard. (**B**): 2-pt shooting after curl-cut with the angle of 0° from the backboard.

**Figure 2:**
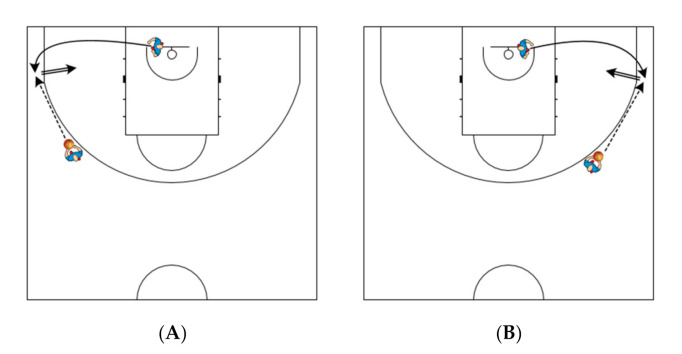
(**A**): 3-pt shooting after curl-cut from the left side. (**B**): 3-pt shooting after curl-cut from the right side.

Before each testing session, the players did a standardized warm-up, consisting of jogging with dribbling, shooting, lay-ups, and dynamic stretching. After the warm-up, players shot freely for 5 min—pull-up jump shots or catch-and-shoot jump shots. Each player performed four 2-pt shots from the right side and four 2-pt shots from the court’s left side. The horizontal distance of the two shots on each side of the basket was approximately 4 m with an angle of 0° from the backboard. For the other two shots, the distance was approximately 4.9 m with an angle of 60° from the backboard (“elbow of the paint”). In a 2-pt shooting, player run from the first cone (1.8 m from baseline and 3.5 m from sideline), behind the second (3.75 m from baseline and 3.3 m from sideline) and third cone (5.7 m from baseline and 4.2 m from sideline) (Figure 1A), and vice versa (Figure 1B). Players also perform two 3-pt shots from the spot close to both corners of the court. All shots have been taken after a pass and after the player run around the cones to the marked shooting spot (catch-and-shoot situation). With a 3-pt shooting, the player run from underneath the basket to the corner of the court (see Figure 2). Player shot after a pass from an assistant coach (approximate distance 6.6–6.75 m). If the pass was not accurate (outside the shooting pocket), the player repeated the attempt. There was a rest period of at least 30 s between each attempt on each side. The player first attempted four 2-pt shots from one side of the court, then from the other side, and then moved to a 3-pt shooting where the player first attempted 2 shots from one side and then 2 attempts from the other side. The players have been instructed to shoot directly to the hoop (not a bank shot) with their natural technique as they do in training and during games, and before each measurement, they had several warm-up attempts.

For analysis of selected kinematic variables of jump shots, the MNV BIOMECH Awinda inertial system (Xsens Technologies B.V., Enschede, The Netherlands) was used. The player wore a full-body suit equipped with 17 wireless motion trackers (sampling frequency 60 Hz) to ensure full 3D motion analysis. Detailed movement analysis was done using the MVN Studio BIOMECH software (Xsens Technologies B.V., Enschede, The Netherlands). Calibration of sensors was set in N-pose. If calibration was rated less than successful it is repeated. After calibration and before start of measuring and each shot, subject was pleased to raise arms (approx. 90° and 180°) to assure that system is measuring correctly, sensors are in good position and calibration was indeed successful. If change of values occur during measuring or sensor moves from initial position calibration was repeated. If movement is very fast and long lasting, multiple calibration should be performed for assuring correct data. Reprocessing of data after recording reduce errors of sensor positioning. Although measuring and data extraction is faster than previous methods, it is very important to be consistent in correct positioning of body segments and sensors. Calibration lasts more than 30 s which can significantly affect time and measurement protocol if there is need of multiple trials. Subject is standing under the basket pointing toward corner (3-pt shot). X-axis represents anterior-posterior movement regard to basket. Because the moment when the ball was received and released from the hand could not be identified by the software MVN Studio BIOMECH, the digital cameras Garmin VIRB ULTRA 30 (Garmin International, Inc., Olathe, KS, USA) were used (sampling frequency 60 fps). The video was used for qualitative purposes. There was no synchronization between camera and Xsens. To measure other parameters, i.e., the ball’s entry angle (when the ball is approaching the rim) and the player’s contact time with the ball, the 94fifty ball (InfoMotion Sports Technologies Inc., Dublin, OH, USA) was used. Ball contains nine accelerometers inside which can detect force (a 360-degree view of it) and speed, ball rotation and ball arc. Ball has a data transmission time of 100 milliseconds. The validity and reliability of 94fifty ball were investigated in a studies by Abdelrasoul et al. [13] and Rupčić et al. [14]. 

### 2.3. Variables

During the jump shot, selected kinematic and physical parameters were observed: (a) center of mass when catching the ball (cm); (b) center of mass minimum with the ball (Z-axis—vertical direction) (cm); (c) shoulder angle at ball release (SA) (°); (d) entry angle of the ball when approaching the rim (EA) (°); (e) player shooting speed (contact time with the ball) (PSS) (s); (f) center of mass anterior-posterior displacement—when both feet touch the ground (X-axis—towards the basket) (cm). The last-mentioned variable (f) was set only for 3-pt shots. Two variables were computed. The first one was the difference between the center of mass when catching the ball and the center of mass minimum with the ball and was termed (g) center of mass difference Z-axis (CoMDZ) (cm). The second one was the difference between the center of mass when catching the ball and the center of mass anterior-posterior displacement and was termed (h) center of mass difference X-axis (CoMDX) (cm). Variables included in statistical analysis: (c–e), (g), and (h).

### 2.4. Statistical Analysis

An a priori analysis using G*Power software (version 3.1.9.2; Heinrich Heine University Düsseldorf, Düsseldorf, Germany) for ANOVA (using an effect size of 0.25, alpha value of 0.05, and power of 0.80) recommended a sample size of 175. Overall, 573 shots were analyzed, while 381 shots were for 2-pt and 192 shots were for 3-pt. The successfulness of all shots was also recorded and included in the analysis. Descriptive statistics were used, and data are expressed as a mean ± standard deviation. Shapiro-Wilk’s test assessed the normality of distribution, and Levene’s test evaluated the homogeneity of variance. When the normality of distribution or homogeneity of variance was violated, the log-transformation of data for eliminating non-uniformity was used [15]. 

Factorial analysis of variance (ANOVA) (between–between design) was used because it simultaneously analyzes the differences in the dependent variables between groups of subjects and between the effectivity of shots [16]. Therefore, the 4 × 2 design (two-way) was applied, where one main factor was a group of players (U16M, U18M, U16F, and U18F), and the other one was a shot effectivity (successful, unsuccessful). Moreover, the interaction of these two factors on the dependent variable was used because it can combine the effect of the factors on the dependent variable. If ANOVA detected any significant differences, the Tukey HSD post hoc test comparisons were carried out. Mean difference (MD), and 95% confidence intervals for post hoc comparison were also determined. The size of the effect was determined by partial eta squared (η^2^_p_), which is suggested by Lakens [17] and Richardson [18]. Partial eta squared values of 0.01, 0.06, and 0.14 indicated a small, medium, and large effect of the measurement [19]. Differences between 2-pt and 3-pt shots for each category separately were assessed by independent t-test and additionally by Cohen’s d. Cohen’s d values were interpreted as 0.2 for small, 0.5 for medium, and 0.8 for large effect size [19].The level of statistical significance was set at α = 0.05. For all statistical analyses, software IBM SPSS Statistics 24 (IBM Corp., Armonk, NY, USA) and Statistica 13.2 (StatSoft Inc., Tulsa, OK, USA) were used. 

## 3. Results

### 3.1. Differences in Categories in 2-pt Shooting

Table 1 provides descriptive statistics for selected kinematic variables. CoMDZ was higher but insignificant in successful shooting attempts compared to unsuccessful ones (F_1,373_ = 2.4, *p* = 0.12, η^2^*_p_* = 0.006). Significant differences with large effect size in CoMDZ were detected between individual age categories of men and women (F_3,373_ = 14.3, *p* < 0.001, η^2^*_p_* = 0.103). The U18F category shot with the smallest difference in the center of mass displacement. A pairwise comparison of categories showed differences between the categories: U16M vs. U16F, *p* < 0.001, MD = 4.3, 95% CI (2.2, 6.4); between U16M vs. U18F, *p* < 0.001, MD = 5.1, 95% CI (2.7, 7.5); between U18M vs. U16F, *p* = 0.01, MD = 2.5, 95% CI (0.4, 4.7); between U18M vs. U18F, *p* = 0.003, MD = 3.3, 95% CI (0.9, 5.8). For interaction effect, difference was not detected (F_3,373_ = 1, *p* = 0.38, η^2^*_p_* = 0.008).

There was no significant difference between successful and unsuccessful shooting attempts in the variable SA (F_1,368_ = 0.09, *p* = 0.76, η^2^*_p_* < 0.001). SA was almost the same for successful and unsuccessful shots. However, the difference in SA was significant between the individual age categories of men and women with large effect size (F_3,368_ = 15.9, *p* < 0.001, η^2^*_p_* = 0.115). Pairwise comparison of categories using Tukey HSD test showed statistical differences: between categories U16M vs. U16F, *p* < 0.001, MD = 7.0, 95% CI (3.0, 11.0); between U18M vs. U16F, *p* < 0.001, MD = 10.5, 95% CI (6.4, 14.6); between U18M vs. U18F, *p* < 0.001, MD = 7.9, 95% CI (3.2, 12.7). Differences in SA in interaction effect were not significant (F_3,368_ = 0.5, *p* = 0.72, η^2^*_p_* = 0.004).

EA was higher in successful shooting attempts compared to unsuccessful shooting attempts. However, the difference was insignificant (F_1,372_ = 2.7, *p* = 0.1, η^2^*_p_* = 0.007). Significant differences with large effect size were recorded between the observed categories U16M, U18M, U16F, and U18F (F_3,372_ = 13.4, *p* < 0.001, η^2^*_p_* = 0.098). Pairwise comparison of categories showed differences: between categories U16M vs. U18M, *p* = 0.002, MD = 2.6, 95% CI (0.8, 4.4); between U16M vs. U16F, *p* < 0.001, MD = 4.5, 95% CI (2.5, 6.4); between U16M vs. U18F, *p* < 0.001, MD = 4.2, 95% CI (2.0, 6.5). Interaction effect was also insignificant (F_3,372_ = 1.1, *p* = 0.34, η^2^*_p_* = 0.009).

PSS was very similar for successful and unsuccessful shots, with minimal difference. there were no differences identified in shooting speed between successful and unsuccessful shots (F_1,373_ = 1.5, *p* = 0.22, η^2^*_p_* = 0.004). There were no differences between the categories (F_3,373_ = 1.4, *p* = 0.25, η^2^*_p_* = 0.011) or the interaction effect (F_3,373_ = 0.3, *p* = 0.85, η^2^*_p_* = 0.002).

### 3.2. Differences in Categories in 3-pt Shooting

Descriptive statistics of selected kinematic variables for 3-pt shooting are given in Table 2. There were no differences in shooting efficiency (F_1,184_ = 0.4, *p* = 0.51, η^2^*_p_* = 0.02) recorded in the CoMDZ variable between categories (F_3,184_ = 0.3, *p* = 0.84, η^2^*_p_* = 0.05), nor in the interaction (F_3,184_ = 0.2, *p* = 0.87, η^2^*_p_* = 0.04).

The differences in SA were small and insignificant between successful and unsuccessful shooting attempts (F_1,183_ = 1.9, *p* = 0.17, η^2^*_p_* = 0.01). ANOVA detected a significant difference and large effect size between categories (F_1,183_ = 9.3, *p* < 0.001, η^2^*_p_* = 0.13). Pairwise comparison using the Tukey HSD test showed differences: between U16M vs. U16F, *p* < 0.001, MD = 12.1, 95% CI (5.6, 18.6); between U18M vs. U16F, *p* < 0.001, MD = 16.2, 95% CI (9.5, 22.8); between U18M vs. U18F, *p* < 0.002, MD = 10.9, 95% CI (3.3, 18.5). The difference in SA in interaction was not significant and the effect size was small (F_3,183_ = 0.8, *p* = 0.49, η^2^*_p_* = 0.01).

EA was higher in successful shooting attempts compared to unsuccessful shooting attempts. The difference was significant, the effect size indicates a small effect between the average EA values (F_1,183_ = 6.0, *p* = 0.02, η^2^*_p_* = 0.03). The difference between the categories was insignificant in EA (F_3,183_ = 1.9, *p* = 0.13, η^2^*_p_* = 0.03), similarly as for the interaction (F_3,183_ = 0.6, *p* = 0.6, η^2^*_p_* = 0.01).

The PSS variable was lower for successful than for unsuccessful shooting attempts, but the difference was insignificant (F_1,183_ = 0.6, *p* = 0.46, η^2^*_p_* = 0.03). Differences were also noted between the categories and the effect size indicated a medium effect (F_3,180_ = 4.3, *p* = 0.06, η^2^*_p_* = 0.067). No significant differences were identified in the interaction (F_3,180_ = 0.5, *p* = 0.65, η^2^*_p_* = 0.009). Pairwise comparison using Tukey HSD test showed specific differences between the U16M vs. U18F categories, *p* = 0.03, MD = 0.07, 95% CI (0.02, 0.12), and between U16F vs. U18F *p* = 0.02, MD = 0.08, 95% CI (0.02, 0.13). 

In CoMDX, there was no difference between successful and unsuccessful shooting attempts (F_1,182_ = 0.02, *p* = 0.88, η^2^*_p_* < 0.01). There were also insignificant differences between the categories, but the effect size indicates a large effect (F_3,182_ = 2.2, *p* = 0.09, η^2^*_p_* = 0.34). Differences in the interaction were identified as insignificant (F_3,182_ = 1.6, *p* = 0.2, η^2^*_p_* = 0.03).

### 3.3. Differences between 2-pt and 3-pt Shooting

**Figure 3:**
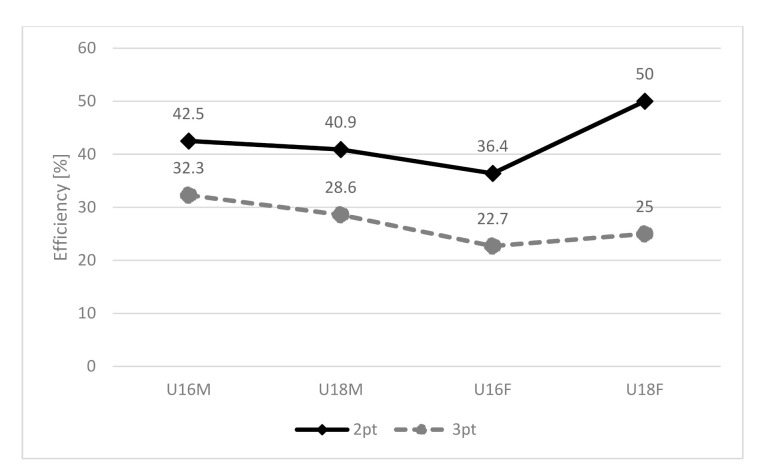
Efficiency of 2-pt and 3-pt shooting in individual categories.

Differences between 2-pt and 3-pt shooting are presented in Table 3. The efficiency of 2-pt and 3-pt shooting of individual categories is depicted in Figure 3. Significant differences and large effect size between 2-pt and 3-pt shooting in U16M were in the variables of CoMDZ (*p* < 0.001; d = 0.83), EA (*p* < 0.001; d = −1.35), and PSS (*p* < 0.001; d = −119). There were no differences found in SA in all categories (*p* > 0.05). In the U18M category, there was difference between 2-pt and 3-pt shooting in the variables CoMDZ, EA, PSS (*p* < 0.001; d = 0.63, −2.23, −1.01). In the U16F category, the difference between 2-pt and 3-pt shooting was not significant in the variables CoMDZ and SA (*p* > 0.05). There was difference in the variables EA and PSS (*p* < 0.001). In the U18F category, the difference between 2-pt and 3-pt shooting was significant only in the EA variable (*p* < 0.001; d = −2.57. In the other variables (CoMDZ, SA, PSS), the difference was not significant (*p* > 0.05).

## 4. Discussion

The main finding of the study is that female and male basketball players use different shooting techniques. We found out differences in 2-pt shooting in variable CoMDZ, SA, and EA between male and female categories. In the 3-pt shooting, we found out differences between male and female categories in variable SA and based on the effect size in variable CoMDX. Player shooting speed was higher in all categories (except U18F) when shooting for 3-pt. Therefore, our hypothesis that differences in selected kinematic variables will be observed between 2-point and 3-point shots regarding different categories and success of the shots can be partially accepted having in mind that we did not found differences between successful and unsuccessful shots.

### 4.1. Differences in Categories in 2-pt Shooting

We did not find differences between successful and unsuccessful 2-pt shots in CoMDZ, SA, EA, and PSS based on statistical analyses. These findings correspond to the results of Uygur et al. [20] who also did not find differences in selected kinematic parameters (elbow, trunk, knee, and ankle joint angles) for free throw shooting between successful and unsuccessful attempts. In the CoMDZ variable, differences were identified between the individual categories (U16M vs. U16F, U16M vs. U18F, U18M vs. U16F, U18M vs. U18F). In male basketball players, in both categories, the difference between the center of mass displacement when catching the ball and the lowest point of the center of mass was greater than that of female players. The reason could be that male players release the ball during a jump shot from a greater height, and therefore a greater power impulse must be given to the lower limbs than it is for the female players [21]. On the other hand, the ball release height also affects the angle at which the ball is released [22]. A larger SA at the time of ball release was found in the male categories. The differences were between U16M vs. U16F, U18M vs. U16F, U18M vs. U18F. The lower SA in female players may be caused by the maximum flexed position adopted during the preparatory phase, which is also related to the lower entry angle of the ball [23]. In the male categories, SAs are presented lower than in the studies of Okazaki and Rodacki [8], Okazaki and Rodacki [24], Rojas et al. [10], namely 118.6°, 119.06°, 136.95°, respectively. In the above research, it was a set shot, except for Rojas et al. [10], where the shooting was carried out after a pass. The differences may have been due to higher age and greater experience of the players, as the mentioned studies involved adult players at the age of about 25 (except for Khlifa et al. [25]. This statement was also confirmed by Button et al. [26] and found greater consistency in kinematic patterns of free throws for players who had more experience in competitive games. Okazaki and Rodacki [24] report SA in 12-year-old children 102.54°, which confirms our assumptions. Female players shot with a lower SA as stated by Elliott and White [23], 113.8°, but we again attribute the difference to age and experience, as they were professional players. SA may also depend on the presence of a defender, as confirmed by Rojas et al. [10], and players in the presence of a defender shot with a larger SA than without the defender.

In the variable EA, differences were recorded between category U16M and categories U18M, U16F, and U18F. Male players shot with more EA than female players. However, these EA values are lower compared to Rupčić et al. [14], where EA was 42.28° in male players U16. The difference may also be that in the mentioned research, players shot from distances of 6 m and 6.75 m, and the given value is the average of shots from both distances. Nevertheless, the average angles of the U18M, U16F, and U18F categories appear to be too small, so it is possible that players in these categories had to shoot with more rotation of the ball to increase the chance of a successful shot. The entry angle of the ball entering the basket is considered one of the main criteria for a successful shot. As the EA of the ball increases, the width of the basket increases [8,27]. Therefore, the smaller EA is probably related to the smaller SA, which was observed in all categories. Based on the results, the lower EA is characteristic of young players, where the parabola of the trajectory of the shot ball is lower. We assume that reducing the rim diameter can be a good tool for young players, where players would have to shoot with a larger SA, and thus their EA would also increase. For example, in a study by Khlifa et al. [25], a reduction rim diameter of 0.35 m (regular rim diameter is 0.45 m) was used, where the minimum EA for the direct shot was 44.48°.

No differences between categories were found in the PSS. Male and female players shot at about the same time from catching the ball to the release phase of the ball. PSS corresponds to the results of studies by Gorman and Maloney [28], van Maarseveen [29] and Oudejans [12]), Rojas et al. [10], and Rupčić et al. [14], where the PSS was ~ 0.82 s, 0.896 s, 0.86 s, and 0.82 s, respectively. Okazaki and Rodacki [8], Okazaki and Rodacki [24], and Podmenik et al. [30] reported slightly lower PSS: 0.74 s, 0.77 s, and 0.73 s, respectively. Shots in the researches, Okazaki and Rodacki [8,24] and Podmenik et al. [30] were not performed after the previous pass, which could have caused the difference just mentioned. As a result, players need more time to catch and shoot the ball than when they are already holding the ball in their hands.

From a practical point of view, PSS is significant because the shorter the time interval from catching the ball after passing to the moment the ball is released towards the basket, the more difficult it is for the defender to block a jump shot [10,31]. The PSS will also be affected by using the ground reaction force. If players cut and get into a catch and shoot situation, they need to transform the horizontal movement into a vertical one by using the ground reaction force in best possible way. Krause et al. [32] recommend that if a player wants to make a jump shot as quickly as possible, he should get into a low center of mass position with slightly bent lower extremities before receiving the ball.

### 4.2. Differences in Categories in 3-pt Shooting

There were no differences between the successful and unsuccessful 3-pt shots in the variables CoMDZ, SA, PSS, CoMDX. Differences between successful and unsuccessful attempts were recorded in the EA parameter. The EA of the ball was on average 1.6° greater in successful attempts than in unsuccessful ones (44° vs. 42.4°). In both cases, however, EA can be considered sufficient, with unsuccessful shots having a lower release velocity of the ball, which is associated with higher shooting successfulness [22,33].

When comparing the differences in CoMDZ, there were no differences noted between the individual categories. It could be because young male and female players release the ball at a lower vertical point when shooting for 3-pt than for 2-pt. In SA, differences were observed between the categories U16M vs. U16F, U18M vs. U16F, and U18M vs. U18F. The difference in SA between the categories may have been due to a greater flexion in the elbow joint in female players, a forward movement of the dominant foot, and a greater horizontal shift in the center of mass [23,34]. The SA is similar in the male categories compared to Okazaki and Rodacki [8] and [7], where the SA was 117.5° and 123.3°, respectively. In these studies, however, players shot from a distance of 6.4 m. No differences were found between the individual categories in the variable EA. All players shot with about the same EA. The lowest EA was achieved in the U16F category. A similar EA is reported by Dobovičnik et al. [35], where it reached an average value of 41.58° for male players U18, which is in line with our findings. PSS was different in each category. Differences were recorded between the categories U16M vs. U18F and U16F vs. U18F. The U18F players achieved the fastest shooting. The PSS is larger than reported by Dobovičnik et al. [32], Okazaki and Rodacki [8], Podmenik et al. [30], and Gorman and Maloney [25]: 0.79 s, 0.67 s, 0.64 s and ~ 0.81 s, respectively. In the studies of Okazaki and Rodacki (2012), and Podmenik et al. [27], players performed a jump shot while already holding the ball in their hands. In the study of Dobovičnik et al. [32] and Gorman and Maloney [28], players stood in place waiting for a pass, after which they immediately made a jump shot. In our case, the players had to make a curl cut for the ball and only then received a pass, after which they could shoot. It reflects that players need more time to coordinate their movement after the previous cut and subsequent pass. It is a more demanding motor task, which is greatly influenced by the previous activity (curl cut).

No difference was found between the categories in CoMDX, but the effect size indicated a large effect. However, when we look at the results, the female players shot with a larger displacement of CoM in the horizontal direction than the male players. The reason may be less power in lower and upper extremities. The consequence is then the forward movement of the dominant foot when stopping, and thus a larger displacement of the CoM in the horizontal direction occurs, which corresponds to Elliott [34]. Podmenik et al. [30] reported CoM displacement toward the basket with a distance of 6.75 m at the level of 30 cm. Elliott and White [23], and Okazaki and Rodacki [8] found a slightly higher CoM horizontal displacement from a distance of 6.4 m, namely, 40.4 cm and 50.3 cm, respectively. The differences concerning the studies mentioned above could have been due to the curl cut, which players had to perform before a pass. It means that after the curl cut, players try to transform the horizontal movement into a vertical one and minimize the displacement toward the basket.

### 4.3. Differences between 2-pt and 3-pt Shooting

Differences between 2-pt and 3-pt shooting were in the categories U16M and U18M in the variables CoMDZ, EA, PSS. In the categories U16F and U18F, there were differences in the variables EA and PSS (only for U16F).

It can be speculated that male players do not need to use as much power in the lower limbs for a 3-pt shot as in a 2-pt shot. It means that there was less countermovement in the 3-pt shot than in the 2-pt shot, which probably also caused to reduce the jump height and earlier release of the ball than in the jump height peak [7]. These findings are consistent with Elliott [33,34], and Okazaki and Rodacki [8], who argue that premature ball release at the time before reaching the jump height peak and lower jump provides the use of some of the jump energy to optimize the ball release impulse. These statements are also characteristic of the jump shot of female players. Interestingly, SA was slightly higher for 3-pt shots for male players and lower for female players. However, the differences were insignificant. Elliott and White [23], Miller and Bartlett [7], and Okazaki and Rodacki [8] report a reduction in shoulder flexion with increasing horizontal distance. Entry angle of the ball when approaching the rim increased for all categories in 3-pt shooting compared to 2-pt shooting. The EA may result from a larger release angle along with the release height and release velocity of the ball [22,35]. As the EA increases, so does the chance of success, and thus the width of the basket [8,27]. On the other hand, Erčulj and Supej [9] point to the negative effect of increasing release angle on shooting success. With increasing release angle, the flight trajectory of the ball also lengthens, making it more challenging to achieve the required accuracy. With a higher release angle, a larger release velocity of the ball and thus a larger force impulse is required [7]. Players have to reorganize the coordination of the body segments to meet the demands of the new task [8]. As a result, there may be a more significant failure in 3-pt shooting, as evidenced by our results (Figure 3).

PSS was different between 2-pt and 3-pt shooting in the U16M, U18M, and U16F categories. Players in these categories needed more time after the pass to release the ball. We assume that the U18F players had approximately the same values in the PSS due to maturity status and their more stable coordination of movements. Players of other categories are still in the developmental process (e.g., growth, hormones), and their coordination of movements may not be at the same level as in adults. The differences found between 2-pt and 3-pt shooting may have occurred because players needed to give more release velocity of the ball and more force impulse [7]. In accordance with Dobovičnik et al. [35], players performed excessive movements with hands before releasing the ball to optimize the force impulse. In real game conditions, any excessive movement can slow players’ shooting speed, giving the defender a better chance to close out or block a shot. In this point of view, Vencurik and Nykodym [36] determine that with increased defensive pressure, the chances for a successful shot decreases. The results of this study confirm the differences in some kinematic variables with other research. In this, we agree with the statement of Okazaki et al. [22] that the performance of skills could probably influence shooting performance before shooting, such as catching a pass and making a cut.

Major limitation in the current study is the lack of confounding variables such as morphological factors. Therefore, future studies should include morphological factors due to possible influence on the results. Nevertheless, this study’s novelty and uniqueness is in the documented selected kinematic variables of the jump shot after making a cut and receiving a ball. For more precise and general conclusions, more subjects and more measured shots are needed. Further research in this area should focus on assessing the kinematic and physical parameters of the jump shot in situations that are more similar to real game conditions (e.g., pull-up jump shots, catch-and-shoot situations after a cut, defended shots).

## 5. Conclusions

Our hypothesis could be accepted having in mind our results concerning categories and success of the shots. The current results show that female and male basketball players used different shooting techniques. Players in male categories shot with a higher center of mass difference in the vertical direction, with a higher release shoulder angle, and with a higher entry angle of the ball. 

Moreover, the entry angle of the ball increases in all categories when shooting for 3-pt. It means that players need more time for 3-pt shots after receiving a pass when compared to 2-pt shots. Therefore, the players are using excessive movements to optimize the shooting technique when shooting for 3-pt. Basketball coaches and players should work to minimize the kinematic differences between 2-pt and 3-pt shooting to increase the successfulness of shooting from longer distances. 

## Figures and Tables

**Table 1:** Descriptive statistics of selected kinematic variables for 2-pt shooting according to efficiency and category.

		CoMDZ (cm)	SA (°)	EA (°)	PSS (s)
Efficiency	Category	M ± SD	M ± SD	M ± SD	M ± SD
**Successful**	U16M	6.5 ± 6.2	110.9 ± 10.4	35.0 ± 6.0	0.83 ± 0.09
	U18M	5.1 ± 5.8	113.8 ± 11.4	32.2 ± 3.9	0.83 ± 0.1
	U16F	2.5 ± 6.8	102.7 ± 7.7	31.6 ± 5.4	0.85 ± 0.09
	U18F	0.4 ± 6.4	104.3 ± 13.3	31.8 ± 5.5	0.85 ± 0.11
	Total	4.2 ± 6.6	108.9 ± 11.6	32.9 ± 5.4	0.84 ± 0.1
**Unsuccessful**	U16M	5.1 ± 5.1	109.5 ± 13.8	34.9 ± 5.8	0.84 ± 0.09
	U18M	3.1 ± 5.2	113.4 ± 11.4	32.5 ± 5.2	0.84 ± 0.1
	U16F	0.8 ± 5.7	103.3 ± 7.5	29.7 ± 5.1	0.87 ± 0.08
	U18F	1.2 ± 6.4	107.0 ± 10.7	29.6 ± 6.4	0.85 ± 0.11
	Total	2.9 ± 5.7	108.8 ± 12.0	32.2 ± 5.9	0.85 ± 0.09
**Total**	U16M	5.7 ± 5.6 ^bc^	110.0 ± 12.5 ^b^	34.9 ± 5.9 ^abc^	0.84 ± 0.09
	U18M	3.9 ± 5.5 ^bc^	113.6 ± 11.4 ^bc^	32.3 ± 4.7	0.84 ± 0.1
	U16F	1.4 ± 6.1	103.1 ± 7.6	30.4 ± 5.2	0.86 ± 0.08
	U18F	0.6 ± 6.3	105.6 ± 12	30.7 ± 6.0	0.85 ± 0.11
	Total	3.5 ± 6.1	108.8 ± 11.8	32.5 ± 5.7	0.85 ± 0.1

Legend: M—mean, SD—standard deviation; ^a^—difference when compared to U18M, ^b^—difference when compared to U16F, ^c^—difference when compared to U18F.

**Table 2:** Descriptive statistics of selected kinematic variables for 3pt shooting according to efficiency and category.

		CoMDZ (cm)	SA (°)	EA (°)	PSS (s)	CoMDX (cm)
Efficiency	Category	M ± SD	M ± SD	M ± SD	M ± SD	M ± SD
**Successful**	U16M	1.8 ± 5.2	114.8 ± 16.2	43.7 ± 4.8	0.96 ± 0.12	18 ± 12.5
	U18M	0.4 ± 4.8	115.2 ± 14.6	43.1 ± 4.2	0.9 ± 0.04	13.4 ± 11.1
	U16F	2.2 ± 6.3	103.4 ± 10.3	44.3 ± 4.3	0.93 ± 0.08	21.6 ± 13.9
	U18F	1.3 ± 4.2	111.4 ± 11.8	46.7 ± 4.3	0.89 ± 0.12	15.9 ± 10.2
	Total	1.4 ± 5.1	112.3 ± 14.5	44.0 ± 4.5 ^†^	0.93 ± 0.1	17 ± 12.1
**Unsuccessful**	U16M	0.2 ± 6.8	111.6 ± 14.5	42.6 ± 4.6	0.95 ± 0.08	13.4 ± 11.1
	U18M	0.7 ± 5.4	117.3 ± 11.6	42.4 ± 4.1	0.93 ± 0.06	14.4 ± 14.8
	U16F	1.3 ± 6.5	99.7 ± 10.6	41.1 ± 4.9	0.97 ± 0.06	18.4 ± 9.3
	U18F	0.9 ± 4.6	103.9 ± 9.5	44 ± 4.3	0.88 ± 0.11	24 ± 13.2
	Total	0.7 ± 6.0	109.1 ± 13.8	42.4 ± 4.5	0.94 ± 0.08	16.5 ± 12.7
**Total**	U16M	0.7 ± 6.4	112.6 ± 15 ^a^	43 ± 4.6	0.95 ± 0.1 ^b^	14.8 ± 11.7
	U18M	0.6 ± 5.2	116.7 ± 12.4 ^ab^	42.6 ± 4.1	0.92 ± 0.06	14.1 ± 13.8
	U16F	1.5 ± 6.4	100.5 ± 10.5	41.9 ± 4.9	0.96 ± 0.06 ^b^	19.1 ± 10.4
	U18F	1.0 ± 4.5	105.8 ± 10.4	44.7 ± 4.4	0.88 ± 0.11	22 ± 12.8
	Total	0.9 ± 5.8	110. ± 14.1	42.9 ± 4.6	0.93 ± 0.09	16.7 ± 12.5

Legend: M—mean, SD—standard deviation; ^a^—difference when compared to U16F, ^b^—difference when compared to U18F, ^†^—difference when compared to unsuccessful shots in total.

**Table 3:** Differences in kinematic variables between 2-pt and 3-pt shooting in individual categories.

Category	CoMDZ (cm)		SA (°)		EA (°)		PSS (s)	
	*p*	*d*	*p*	*d*	*p*	*d*	*p*	*d*
U16M	< 0.001	0.83	0.570	−0.09	< 0.001	−1.35	< 0.001	−1.19
U18M	< 0.001	0.63	0.107	−0.26	< 0.001	−2.23	< 0.001	−1.01
U16F	0.913	−0.02	0.111	0.31	< 0.001	−2.04	< 0.001	−1.32
U18F	0.755	0.08	0.956	−0.01	< 0.001	−2.57	0.248	−0.27

## Data Availability

The data presented in this study are available on request from the corresponding author. The data are not publicly available due to its huge size and participants’ privacy protection.
